# A two-phase hybrid clustering framework exploring transitional activities in HAR

**DOI:** 10.1007/s44163-025-00503-6

**Published:** 2025-08-31

**Authors:** Martin Woo, Ahmed A. Harby, Farhana Zulkernine, Hanady M. Abdulsalam

**Affiliations:** 1 School of Computing, Queen’s University, ON K7L 3N6 Kingston, Canada; 2 Information Science Department, Kuwait University, 13060 Kuwait City, Kuwait

**Keywords:** Stream clustering, Autoencoders, Human activity recognition, CNN

## Abstract

Human Activity Recognition (HAR) using data streams from wearable sensors is challenging due to high data dimensionality, noise, and the lack of labeled data in unsupervised settings. Our prior work proved that traditional clustering models, which achieve state-of-the-art performance on simulated datasets, perform poorly on time-series numeric sensor data. This paper explores different autoencoder (AE) architectures to extract latent features with reduced dimensionality from streaming HAR datasets, which is then clustered using a clustering model to identify different activity patterns. Since the vanilla AE has shortcomings in learning distinguishing data patterns from spatio temporal time-series sensor data, we leverage the vanilla AE with convolutional, long-short term memory (LSTM), and a combination of convolutional and LSTM layers in multiple design phases. We apply supervised learning to train a superior spatio-temporal feature extraction AE model. Using the data features extracted by the trained AE, we train a clustering model with unsupervised learning approach. Our end-to-end integrated hybrid convolutional AE+LSTM feature extractor and K-Means clustering model achieves state-of-the-art clustering accuracy of up to 0.99 in terms of Normalized Mutual Information (NMI) and Adjusted Rand Index (ARI) scores for MobiAct and UCI HAR datasets, improving clustering performance by over 50% compared to previous methods. Further improvements are achieved through rigorous experimentation and advanced data preprocessing methods. We also present a visualization of the clusters, which explains the transitional activity patterns in the overlapping parts of the clusters.

## Introduction

Research on Human Activity Recognition (HAR) focuses on extracting signature data patterns from different types of spatio-temporal data streams, such as numeric time-series, signal, video, and point cloud data, to identify human motions or activities. HAR can be applied to rehabilitation and healthcare [1–4], fall detection [5–7], surveillance [8–10], remote senior care [1, 11–13], and physiological studies [14]. Identification and analysis of fall patterns or transitional states to fall can provide a deeper understanding of the motion patterns to suggest preventive actions and allow early notification to lower the risk of fall and sustaining injuries.

Smartphones and smart wearable devices, equipped with built-in sensors, have facilitated data collection and helped improve the accuracy of HAR [15, 16]. However, human movements have a natural flow, making it challenging for models to discern the boundary between the end of one action and the beginning of another. Considering a sequence starting from standing up to sitting down can be broken down into sub-movements: (1) Standing, (2) Transition from standing to sitting, and 3) Sitting. Typically, the transition phase would not be considered a distinct action by sensor systems because transitional movements are brief and numerous, making them difficult to classify. Consequently, HAR datasets often lack labels for these transitional movements, leading to the question of whether such transitions can be accurately detected.

Many existing studies on HAR focus primarily on supervised learning methods [15–18], based on IoT data from smartphones and smartwatches [14, 15]. While unsupervised clustering has been applied to image data [19, 20], not much work exists that apply deep learning based clustering models to time-series IoT data [21]. Furthermore, detecting transitions between activities with time-series data is a difficult problem because of the brief duration and overlapping nature of the signals. Earlier work, such as Bulling et al. [22] and Ravi et al. [23], pointed out that traditional classification techniques struggled to recognize the transitional activities, and often led to misclassifications. Hammerla et al. [24] emphasized the effectiveness of temporal models such as long short-term memory(LSTM) to capture long-term dependencies. Hybrid approaches that combine a convolutional neural network(CNN) and LSTM [25] have also been effective in capturing both spatial and temporal features.

In this work, we specifically address the challenges in clustering time-series numeric sensor data for HAR and identifying transitional activity patterns. In our previous work [26], we explored several state-of-the-art statistical stream clustering algorithms, which demonstrated high accuracy on simulated benchmark data streams. When applied to time-series numeric HAR data. The models performed poorly due to their inability to process and extract distinguishing patterns from complex and noisy time-series numeric HAR data with overlapping transitional activity patterns and/or constantly evolving concepts. We hypothesize that an efficient computational method capable of processing and reducing high volume and high-dimensional data (e.g., HAR data is collected every millisecond or second from multiple sensors) while preserving significant data features would lead to a good solution for clustering HAR data streams.

Autoencoders (AE) have demonstrated great success in extracting important data features in real-time through comparatively simpler deep learning architecture and can effectively reproduce the pseudo original data patterns [27]. Our findings reveal that the vanilla AEs struggle to learn representative features from the noisy multidimensional *time-series numeric sensor* data, which affects the clustering performance. Therefore, we explore several AE models to extract high-quality data patterns for clustering time-series numeric HAR data, considering the difficulty of labeling high-volume sensor data. In the end, we do use a combination of supervised and unsupervised training approaches for training the end-to-end pipeline, which consists of a feature extractor and a clustering model. We use the AE models as a feature extractor and train them using a supervised approach. We present an incremental design approach, where based on the results at each phase, we gradually improve the AE feature extractor. Visualization of the clusters using Tree Map(TMAP) allows for intuitive exploration and understanding of the underlying clusters and transitional activity patterns.

### Contributions

The contributions of this work are as follows:

- We propose an end-to-end hybrid data analytics pipeline to cluster complex high-dimensional numeric time-series data for HAR. We specifically present a multi-phase model design and validation approach where based on the results of each phase, we apply further improvements to our end-to-end pipeline to increase the accuracy of clustering HAR data.
- In each phase, the pipeline uses a *feature extractor* component with a *clustering model*. We improve the overall clustering performance in each phase by enhancing the feature extractor model in terms of architecture and training, and using different data preprocessing techniques to explore both spatial extractors and temporal features and established clustering models, we implement 4 different types of feature extractor models: (i) DEC with a simple AE, (ii) 2D-CNN with and without an AE, (iii) LSTM AE, and (iii) a hybrid Conv AE+LSTM. We illustrate the impact on the overall clustering results through rigorous experimentation. The CNN, LSTM, and hybrid extractors are first trained using a supervised method. Then the clustering model is trained using an unsupervised method with data features extracted by the trained models. Further enhancements are demonstrated with additional temporal data preprocessing techniques.
- Performances of deep embedded clustering(DEC), fast incremental shared nearest neighbor hierarchical density-based clustering(FISHDBC), and wiSARD for clustering data streams(WCDS) are compared as a baseline to that of the integrated clustering pipeline composed of the different data extractors with a K-Means clustering model. The results demonstrate that our hybrid Conv AE + LSTM extractor with K-Means achieves the state-of-the-art performance on benchmark HAR datasets, with Normalized Mutual Information (NMI) and adjusted Rand Index (ARI) scores of up to 0.99 to help for real-time HAR applications. The additional temporal data processing methods to learn advanced temporal features achieve further improvements at an added resource cost.
- Overlapping clusters demonstrate transitional activities. Improvements in clustering help to untangle their complexities, and the clusters explain which of the activities have overlaps and why. We provide a visualization of the clusters and the data to demonstrate the complexities of the data features that explain why traditional clustering models, which perform great with synthetic data, fail to obtain good results on multidimensional HAR time-series data.

The rest of the paper is organized as follows. The related work is presented in Sect. 2. Section 3 outlines the methodology and models employed in our experiments. Details about the implementation and experimental setup are provided in Sect. 4. Section 5 presents the results and discussions. A visualization of the clusters is provided in Sect. 6. Finally, we conclude the paper in Sect. 7.

## Related work

### Feature extraction

Recent advances in HAR have been largely driven by deep learning architectures that enable scalable, robust feature extraction from multivariate sensor streams. CNNs, originally developed for image data, were adapted to one-dimensional time-series HAR signals to exploit local spatial dependencies [28–30]. Munzer et al. [31] demonstrated the effectiveness of sensor fusion through CNN-based architectures, while Chen et al. [32] reported 96% classification accuracy on the MobiAct dataset using a CNN-only pipeline. Sani et al. [33] further improved generalization by incorporating frequency-domain features into CNNs.

To capture temporal dependencies, LSTM networks and their variants were integrated into hybrid models. Thakur et al. [34] combined convolutional autoencoders with LSTM units to enhance temporal feature extraction. Other studies implemented CNN+GRU [35] and CNN+BiLSTM [36] combinations to achieve high classification accuracy across diverse benchmarks. Autoencoders (AEs) also played a central role in learning compressed feature representations. Almaslukh et al. [37] employed stacked AEs for dimensionality reduction, while denoising [38] and sparse [39] variants offered resilience to signal noise and sensor variability.

In parallel, representation learning techniques evolved to address the limitations of supervised architectures. COBRASTS [40] employed constraint-based refinement, and models developed by Tschannen et al. [41] and Xue et al. [42] incorporated recurrent autoencoders and GRU-based contrastive objectives to better encode temporal semantics. In multidimensional contexts, frameworks such as TAPNET [43] and symbolic representations [44] introduced structure-aware transformations to enhance interpretability.

Recent efforts have further emphasized memory and computation efficiency in HAR systems for embedded applications. RepHAR [45] employs structural reparameterization to combine multi-branch networks during training with streamlined CNNs during inference, achieving over 2% accuracy gain and a 72% speedup on Raspberry Pi hardware. Similarly, the CNN-TSFDU-LW model [46] introduces a temporal-spatial decoupling mechanism and layer-wise training to improve feature expressiveness while reducing memory footprint, reaching up to 97.90% accuracy on the UCI-HAR dataset. Additionally, a layer-wise CNN framework with local loss [47] offers better memory reuse compared to traditional global loss models, enabling efficient training for wearable HAR systems without sacrificing performance.

Together, these approaches establish a strong foundation for generalizable and resource-efficient feature extraction pipelines tailored to real-time, sensor-based HAR applications.

### Clustering

While classification-based HAR models have achieved strong performance, they rely heavily on annotated datasets, which limits scalability in real-world scenarios. As a result, clustering methods have gained traction for unsupervised activity recognition. Early models such as DEC [48], FISHDBC [49], and WCDS [50] struggled to account for temporal continuity or transitions in sensor data. To address these deficiencies, Ouyang et al. [51] proposed ClusterFL, a federated learning-based clustering framework that protects user privacy while retaining representation fidelity. More recently, Kumar et al. [52] introduced Cross-Domain Activity Analysis (CDAA), combining GRU-based encoders with clustering objectives to enhance activity recognition across diverse environments.

The field also saw significant growth in contrastive and hierarchical representation learning strategies. SimCLR [53] and TS-TCC [54] improved embedding separability through augmentation-driven contrastive loss, while cluster-based approaches such as SwAV [55] and PCL [56] leveraged prototype-driven constraints to shape latent space geometry. To address coarse-grained prototype refinement, Sharma et al. [57] introduced hierarchical contrastive learning, which was later advanced by Meng et al. [58] through MHCCL. This masked hierarchical strategy improved inter-cluster separation by reducing representation noise and false-negative pairs.

Dataset diversity played a critical role in testing the limits of these models. Traditional HAR benchmarks like PAMAP2 [22] provided controlled environments but lacked real-world complexity. Later datasets, including basketball-specific motion data [59], began to bridge this gap. Most notably, Hoelzemann et al. [60] introduced the *Hang-Time HAR* dataset, which captured wrist-based IMU data from athletes across unstructured and semi-structured scenarios. The dataset incorporated multi-level labeling, intersubject variability, and semi-controlled conditions, making it highly suitable for evaluating clustering and representation learning models under realistic conditions.

Despite these advances, several challenges remain unresolved. Subtle activity transitions often evade model detection, and high-dimensional sensor inputs still obscure interpretability. Our work builds on this body of research by integrating AEs and LSTMs to improve feature representation, while utilizing visualization tools such as TMAP [61] to provide insight into cluster structures. This framework enables unsupervised HAR systems to scale beyond annotation-heavy pipelines while preserving semantic richness in model outputs.

## Methodology

**Fig. 1:**
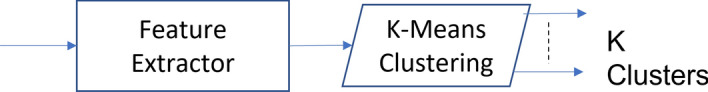
The two-step clustering method

Our approach is composed of two main data processing steps: (1) Feature extraction, and (2) Clustering. High quality data features can provide good clustering accuracy (Fig. 1). Based on the proven success of AEs, LSTM, and hybrid supervised-unsupervised networks in extracting significant data patterns and reducing dimensionality of high volume data as discussed in Sect. 2, we develop and validate four AE-based feature extraction approaches over multiple stages based on the limitations of the previous models. The extracted features are fed into a clustering models, while experimenting and validating the complete end-to-end pipeline to achieve high clustering accuracy. To further understand and evaluate the resulting clusters, we utilize TMAP visualization to intuitively explore high-dimensional data. By creating an interpretable tree structure, TMAP provides insights into the relationships between clusters and allows better assessment of the clusters.

### Data preprocessing

To prepare the data for clustering, we applied the following preprocessing steps consistently across all models:

- *Normalization:* The sensor data was normalized using *MinMax* normalization to rescale values within a uniform range. This ensured that all features contributed equally to the clustering process.
- *Column Removal:* Non-essential columns such as *timestamp*, *rel_time*, and other metadata were removed, focusing on the sensor readings for clustering. The *label* column was retained only for post-clustering evaluation purposes.
- *Data Segmentation:* The data was divided into fixed-size windows using a sliding window approach with no overlap. This segmentation helps to break the continuous time-series data into smaller, manageable chunks for feature extraction.

#### Model-specific preprocessing

The general preprocessing pipeline was further customized to meet the specific needs of each clustering model:

- *FISHDBC preprocessing:* In addition to the general normalization, the data was prepared for distance-based clustering by applying *MinMax* normalization to reduce the range of distances. The unnecessary temporal columns were removed, and only the sensor data was fed into the model.
- *WCDS preprocessing:* The sensor data was normalized to fit within the range of −1 to 1. Due to the streaming nature of WCDS, the data was fed into the model sequentially. As with FISHDBC, only the essential sensor readings were used, while temporal information was discarded.
- *DEC preprocessing:* The DEC model also required *MinMax* normalization, with data scaled between 0 and 1 to match the deep learning architecture of the AE. As with the other models, non-essential columns were removed, leaving only the sensor readings for clustering.

### Feature extraction

AEs learn the representative features from the data as weights in an unsupervised manner using an *encoder* component. The encoder produces compressed latent representations of the input data from its last layer, which lies at the narrow middle part of the AE. This latent representation then goes through a *decoder* component, which is trained to reconstruct the original data as accurately as possible. The optimization function minimizes the reconstruction loss by trying to reduce the difference between the original and reconstructed data.

We design the following four different feature extractors to gradually improve the clustering results: Deep Embedded Clustering AE (DEC) [62], a CNN-based AE, an LSTM-based AE, and a hybrid Conv AE+LSTM. These architectures enable the model to capture high quality data features and learn both spatial and temporal dependencies, which are critical for HAR and identifying transitional activities.

*DEC:* DEC [62] is an AE model designed specifically for clustering. It integrates a clustering layer into the AE, which learns to map the latent space into cluster centroids. The model optimizes the Kullback–Leibler (KL) Divergence loss to fine-tune the clusters during training.

*CNN-based AE:* This model applies 2D convolutional layers to extract better spatial features from the sensor data to improve clustering accuracy. The extracted features are used in two ways: a) directly with a K-Means clustering model, and 2) after reducing the features using a Multilayer Perception (MLP) AE model and then feeding the reduced features to a K-Means clustering model. The model is trained using a supervised learning approach.

*LSTM-based AE:* To handle the temporal aspects of HAR data and improve the clustering accuracy, we utilize an LSTM-based AE, which are particularly well-suited for time-series data. The model learns the sequential patterns in the data using LSTM in the encoder and decoder parts of the AE. The latent features from the AI is used in clustering to better learn transitional activity patterns such as sitting down or standing up.

*Hybrid Conv AE + LSTM:* This hybrid model combines the feature extraction capabilities of both convolutional and LSTM layers to better learn both spatial and temporal dependencies in the data. As in the other models, the latent features from the encoder end are used in clustering. This architecture is particularly effective for recognizing overlapping activities as it balances spatial and temporal feature extraction.

#### Feature extraction strategies and training optimization

Besides the regular data preprocessing methods as described above, other data processing methods were used with the feature extractor models to enhance temporal feature extraction and create a more balanced dataset for model training.

*Time-distributed temporal extraction*: Time series data presents unique challenges due to its temporal dependencies. To capture these dynamics, we applied the Time-distributed wrapper from Keras/TF, which allows non-LSTM layers (e.g., Conv1D, Dropout, Flatten) to process input data across time steps. The data is reshaped, allowing each time slice to be processed independently while maintaining time dependencies across samples.

*Multi-headed temporal extraction*: To further enhance temporal feature extraction, we employ a multi-headed CNN architecture with three heads, each using different kernel sizes (5, 7, 11). These layers process the input sequence at multiple granularity, followed by Dropout, MaxPooling, and Flatten layers. The extracted features are then passed through two BiLSTM layers, followed by Dense layers and Batch Normalization. Recurrent Dropout is applied in the BiLSTM layers to prevent overfitting, and a final Dense layer is used for classification.

*Data upsampling*: We compare the performance of our end-to-end pipeline for slightly more balanced and unbalanced data used in model training. We upsample the classes with fewer data points in some experiments as explained in the results section, using SMOTE (Synthetic Minority Over-sampling Technique) [63].

### Clustering

We explore both traditional and neural network-based approaches namely FISHDBC [49], WCDS, K-Means, and DEC, for the clustering step. We tailor the algorithms to process high-dimensional streaming HAR data efficiently.

*FISHDBC* [49] is an extension of HDBSCAN designed for density-based clustering in evolving data streams. It approximates the Minimum Spanning Tree (MST) to reduce computational complexity while maintaining cluster quality. FISHDBC also uses Hierarchical Navigable Small Worlds (HNSW) for efficient nearest-neighbor approximation, allowing for faster updates and distance calculations. Key parameters such as *min_samples* and *min_cluster_size* control noise sensitivity and cluster size, making it effective for real-time clustering of high-dimensional HAR data.

*WCDS* [50] is a weightless neural network that clusters streaming data using binary representations stored in RAM-like discriminators. The model adapts to real-time data by dynamically updating patterns, preventing outdated data from skewing the results. Normalization techniques are used to address cluster imbalances, and key parameters like $\beta$ (address length) and $\gamma$ (encoding resolution) control the granularity of the clustering.

*K-Means* is a classic clustering algorithm used here to group the latent features extracted by the autoencoders. It assigns data points to clusters based on their proximity to the centroids.

*DEC* is an integrated AE model with a custom clustering layer, as proposed by Xie et al. [48]. DEC transforms data into a lower-dimensional space and optimizes cluster assignments using the Kullback–Leibler (KL) Divergence loss between two probability distributions $P$ and $Q$ using in Eq. 1:

1 $$\begin{aligned} KL(PQ) = \sum _{x \in X} P(x) \cdot \log \left( \frac{P(x)}{Q(x)} \right) \end{aligned}$$

This loss measures how much information is lost when approximating one distribution by another. DEC refines cluster centers iteratively, improving both feature learning and clustering simultaneously.

### Datasets

Models are implemented, trained, and tested with two widely used benchmark HAR time-series sensor datasets, MobiAct [64] and UCI HAR [14] as described in Table 1. The two benchmark datasets that we use to evaluate our framework, were created using data collected from Smartphone sensors, while different participants performed the activities [14]. Table 1 provides a quick summary of the two HAR datasets.

**Table 1:** Summary of the MobiAct and the UCI HAR datasets

Dataset	No. of Subjects (Female/Male)	No. of Activities	No. of features
MobiAct v2.0	66 (16/50)	16	9
UCI HAR	30	6	9

#### MobiAct v2.0 HAR dataset

MobiAct v2.0 dataset [64] represents 16 activities of daily living including common actions such as walking and standing, activities that are fall-like, and activities with sudden movements similar to fall (e.g., jumping, jogging). Version 2.0 also includes scenarios of daily living, which are a series of activities that make up common everyday routines (e.g., leaving home, coming home from work). The scenarios consist of 3,200 trials from 66 subjects with a varying number of activities. Each activity has been recorded using a smartphone with a 3D accelerometer and a gyroscope in addition to orientation data.

**Fig. 2:**
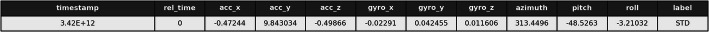
An instance of the MobiAct dataset

Figure 2 shows an example from the MobiAct dataset. Each instance of the data contains 12 columns. The *timestamp* column reflects the time and date as recorded by the Android API. The *rel_time* column is the time of an observation within the trial. The following 9 columns contain the readings of the *x*, *y* and *z* axes for the accelerometer and gyroscope sensor, azimuth, pitch, and roll for the orientation, and the manually assigned activity label (e.g., STD = standing).

#### UCI HAR dataset

The UCI dataset [14] consists of ten-thousand samples pertaining to only six classes of activities, but includes additional sensory information on postural transitions i.e., the motions that are encountered when moving from one body posture to another. The postural data that is collected from the 30 participants have been preprocessed using noise filters and sampled into fixed sliding windows of 2.56 with 50% overlap, for a total of 128 readings per window. Our version of the dataset uses the raw sensor signals captured by the smartphone, instead of the manually constructed feature vectors found in the first iteration of the dataset. Each window contains readings from the smartphone’s embedded 3D accelerometer and gyroscope; the 3 axis from the estimated body acceleration are also included for a total of 9 feature columns.

#### MNIST

The MNIST dataset is a widely-used collection of handwritten digits, consisting of 60,000 training images and 10,000 testing images, each sized 28x28 pixels. It is generally used as a benchmark for evaluating image processing and machine learning algorithms. Due to its simplicity, MNIST is used in one experiment to validate the clustering models and show how the clustering performance differs for MNIST compared to the HAR data.

#### Dataset variants and engineered inputs

In addition to general preprocessing techniques applied to all models, specific variants of the dataset and engineered input configurations were created to explore performance trade-offs and task-specific optimizations.

*MobiAct SLH:* A specialized subset of the MobiAct dataset was constructed to focus on the transitional and high-impact activities: Sitting, Lying, and Heavy Fall. To achieve this, the original dataset was filtered to include only samples from these three classes, and class rebalancing was applied to ensure sufficient representation for each. This subset was used for benchmarking clustering performance under low inter-class variance and high temporal overlap.

*Engineered Reduced MobiAct:* To reduce the input dimensionality and emphasize signal relevance, we derived a reduced dataset variant by selecting only core motion features including accelerometer and gyroscope data. Additionally, basic statistical features such as mean, variance, and maximum values were extracted over each segmented window. These engineered features provide a compact yet expressive input space for both clustering and feature extractor models, enabling improved generalization with fewer parameters.

## Model implementation

We first explain the implementation and training methods of the feature extraction models, and then the clustering models (Sect. 4). An incremental model implementation approach is applied through several phases, where the latter phases are informed by the results of the previous phase. Therefore, after we observed that the DEC model composed of AE-based feature extractor failed to perform well, we gradually extended the AE first with convolutional layers, then with LSTM layers, and finally, with both convolutional and LSTM layers. After the feature extractor AE models are trained using supervised learning, they are used to extract features to feed the clustering model, which is trained with an unsupervised approach.

Two category of models are used in the clustering process, feature extractors and clustering models (see Fig. 1). After implementing the DEC model with unsupervised training, we observed that the performance was poor. Therefore, we tried to apply supervised learning based on the literature to enhance the performance of the feature extractor models (CNN AE, LSTM AE, and the hybrid Conv-AE+LSTM) as described below.

**Fig. 3:**
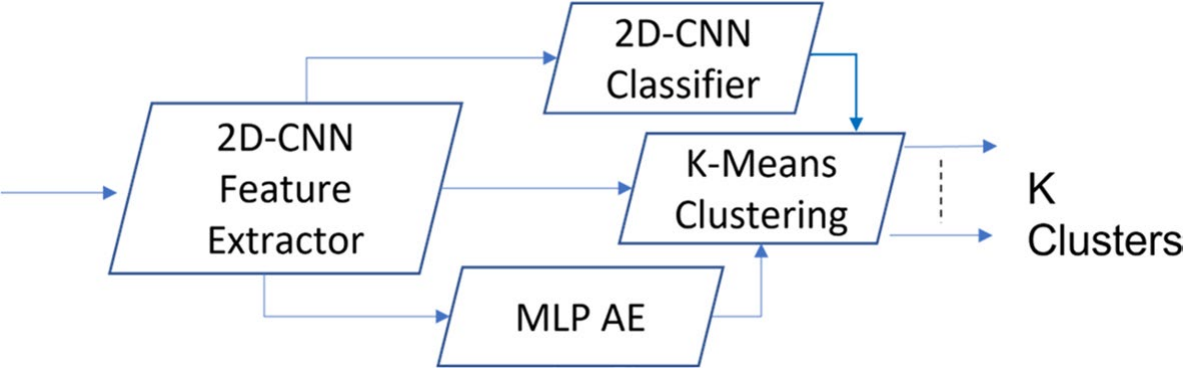
Architecture of the 2D CNN feature extractor with and without the MLP AE model

**Fig. 4:**
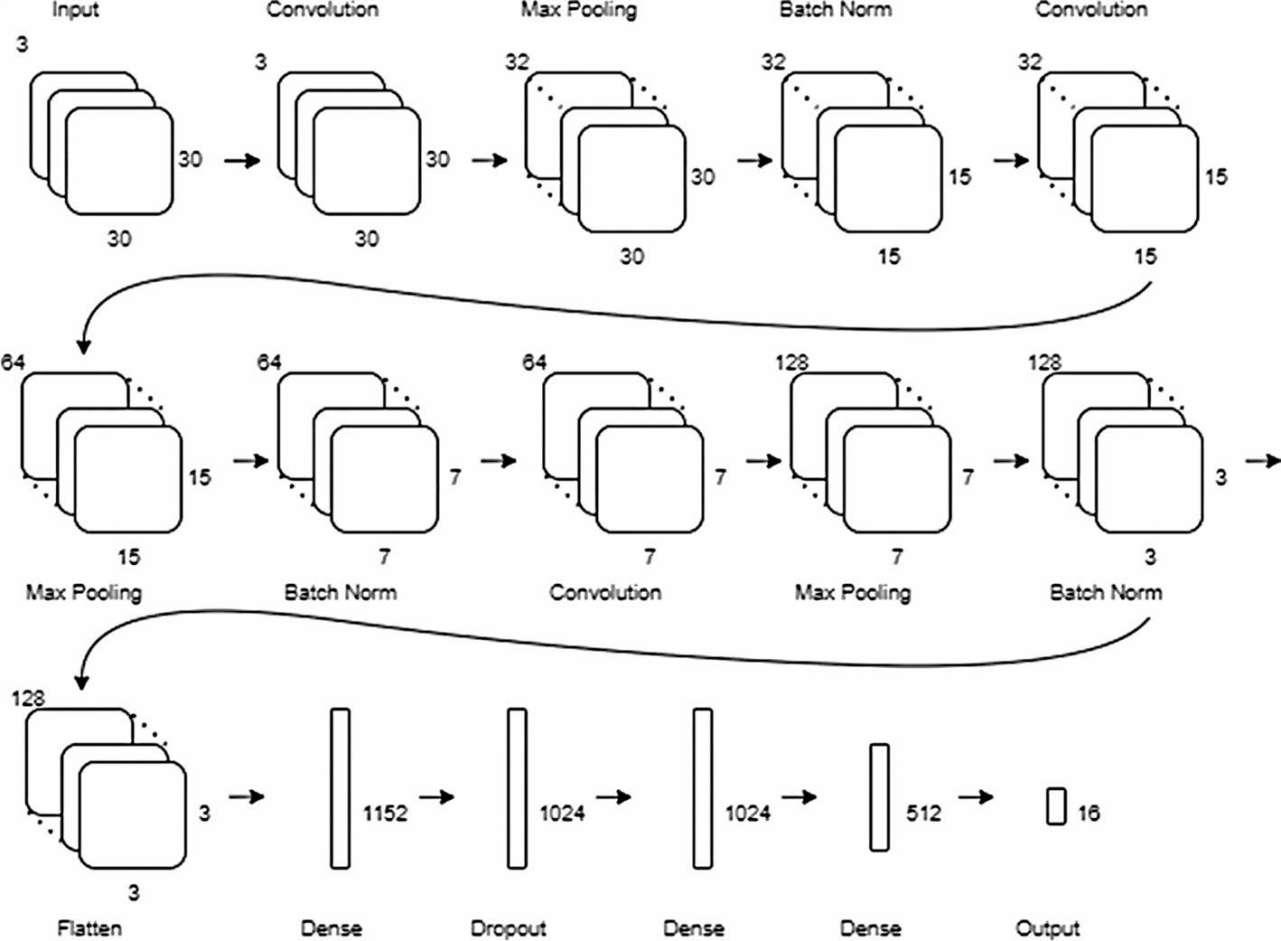
Architecture of the 2D-CNN

### Feature extractor models

We employ a series of deep learning-based feature extractor models to encode high-dimensional sensor data into informative latent representations. These models aim to capture both spatial and temporal dependencies inherent in HAR datasets. Once trained in a supervised setting for classification tasks, the latent features from these extractors are used as input to unsupervised clustering algorithms. This section presents the design, training strategies, and variations of the feature extractors used in our pipeline.

*2D-CNN*: We design a 2D-CNN model with two convolutional blocks, each followed by max-pooling and batch normalization block. We traine the model using *supervised learning* for classification of HAR with categorical cross-entropy loss for 100 epochs. The architecture is shown in Figs. 3 and 4. Features extracted by the trained model are then used to train the K-Means clustering model using using *unsupervised learning*. We implement two versions of this model, with and without an additional MLP AE model, to reduce the features before clustering.

- *Version 1: 2D-CNN without AE* Features from the CNN (before the classification layer) are directly input into the K-Means algorithm for clustering.
- *Version 2: 2D-CNN with MLP AE* Features from the CNN are compressed using a Multi-Layer Perceptron (MLP) AE to reduce dimensionality into a 32-dimensional vector. The compressed features are then clustered using K-Means. The AE uses ReLU activation, with a sigmoid function in the output layer, and is trained for 300 epochs using the Adam optimizer and Mean Squared Error (MSE) loss. The HAR data is transformed from 1D to 2D arrays, representing the X, Y, and Z axes of the accelerometer and gyroscope readings across a 1.5-second window.

**Fig. 5:**
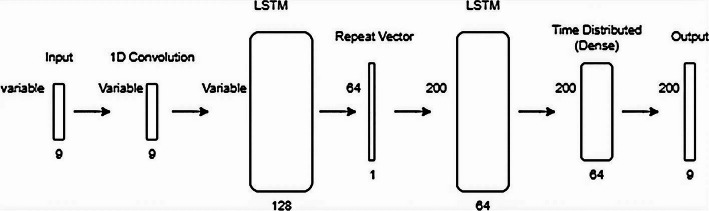
LSTM AE architecture

*LSTM-based AE*: The LSTM-AE is implemented to better learn temporal data dependencies using the full sequence MobiAct, segmented MobiAct, and the UCI HAR dataset. The data is first preprocessed using a 1D convolutional layer and then by a LSTM encoder with two layers (128 and 64 nodes). The decoder mirrors the encoder architecture. The LSTM AE uses ReLU activation, except in the output layer, which uses a sigmoid function. The model is trained for 300 epochs using the Adam optimizer and MSE loss. For model training, the extracted features are passed through an MLP for classification using *supervised learning*. Once trained, the extracted features from the encoder are fed to the K-Means model for clustering. The architecture is shown in Fig. 5.

**Fig. 6:**
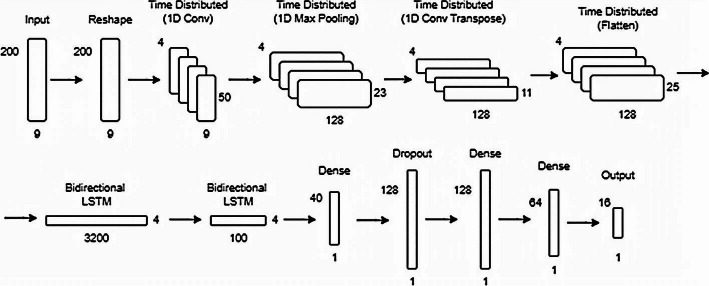
1D ConvAE + LSTM Architecture

*Hybrid AE*: LSTMs are not able to capture spatial features as well as CNNs. Therefore, we integrate a 1D Convolutional AE (Conv) with an LSTM classifier to create a hybrid feature extraction model. The 1D Conv AE extracts features, while the LSTM classifier learns sequential patterns from these features. This hybrid model is trained using *supervised learning* for HAR classification. Once trained, the extracted features during testing are passed through dense layers to the K-Means clustering model. The architecture is shown in Fig. 6.

**Fig. 7:**
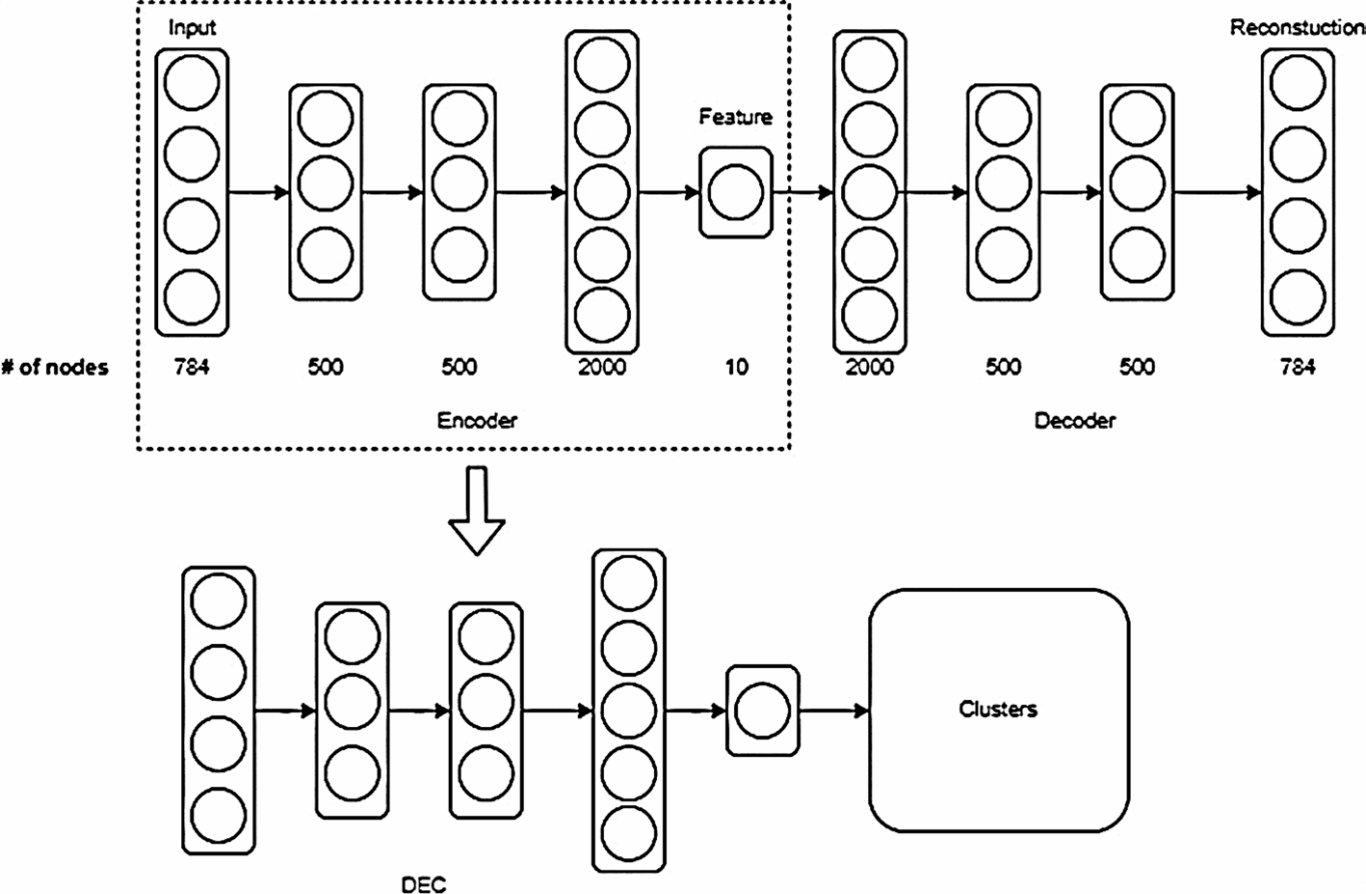
Architecture of the DEC model. The top architecture is the encoder + decoder portion of the DEC model. The bottom architecture is the encoder + clustering head and is the model responsible for the final outputs

### Clustering models

In this study, we implement the following clustering methods to demonstrate comparative performance.

*DEC*: We use the model architecture proposed by Xie et al. [48], which balances depth and speed to optimize performance as shown in Fig. 7. The encoder of the AE model inside the architecture consists of three layers with 500, 500, and 2000 nodes, respectively. The decoder is a mirror of the encoder. The model reduces the input data to 10 latent features, which are then used for clustering. The model is pre-trained using *unsupervised learning* for 300 epochs, and further trained until the stopping criterion of either 20,000 iterations or convergence is reached.

*FISHDBC*: This model is implemented based on [49] as one of the baseline methods.

*WCDS*: WCDS is implemented following the architecture and implementation details in [50] as another baseline method.

*K-Means*: The extracted compressed features from the AE feature extractor are clustered using K-Means. K-Means is implemented using Scikit-Learn. We enhance the clustering performance using a soft assignment method that applies Student’s *t*-distribution as a kernel to measure the similarity between the data points and centroids. This approach allows for more flexible and accurate cluster assignments by improving cluster separation and better handling non-convex distributions in HAR data.

### Experimental setup

The models are implemented and trained on a high-performance computing environment with Intel(R) CPUs and NVIDIA(R) A100 Tesla GPUs. Each AE is pre-trained for 300 epochs to stabilize the model parameters before clustering begins. The clustering algorithms are evaluated using metrics such as Normalized Mutual Information (NMI) and Adjusted Rand Index (ARI) to assess the quality of the clusters. The experimental results are detailed in the following sections.

## Validation and results

The experimental results presented in this section validate the clustering accuracy of the end-to-end pipeline for the MobiAct and UCI HAR datasets. Below, we discuss the results for the classical clustering models including the DEC model, and then for the enhanced AE models. Next, we compare the results for the MobiAct and UCI HAR datasets, and finally with and without the additional data preprocessing methods.

### Baseline clustering performance: FISHDBC, WCDS, and DEC

**Fig. 8:**
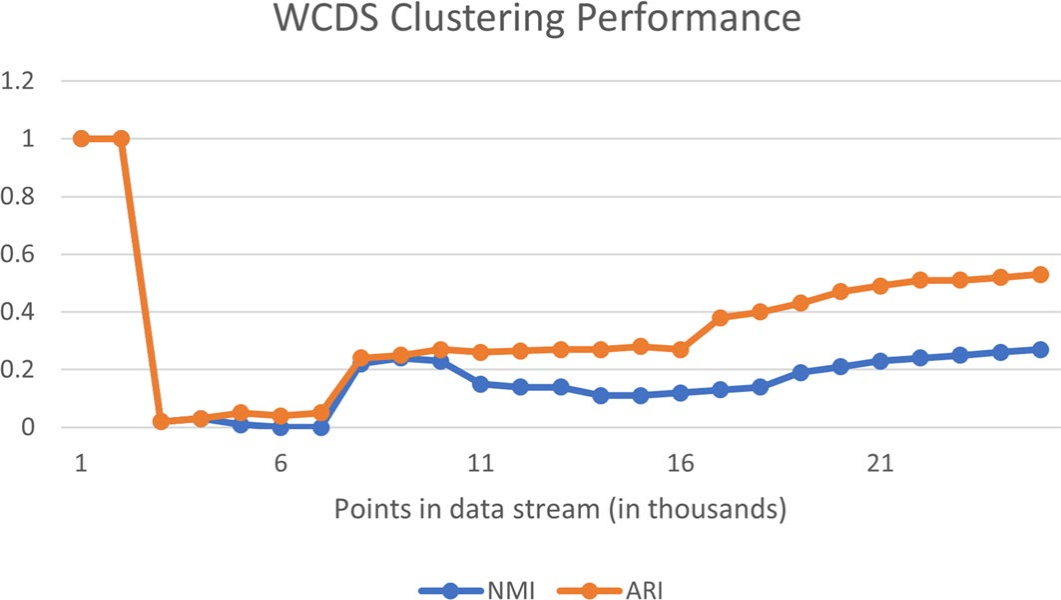
DEC loss during training

**Fig. 9:**
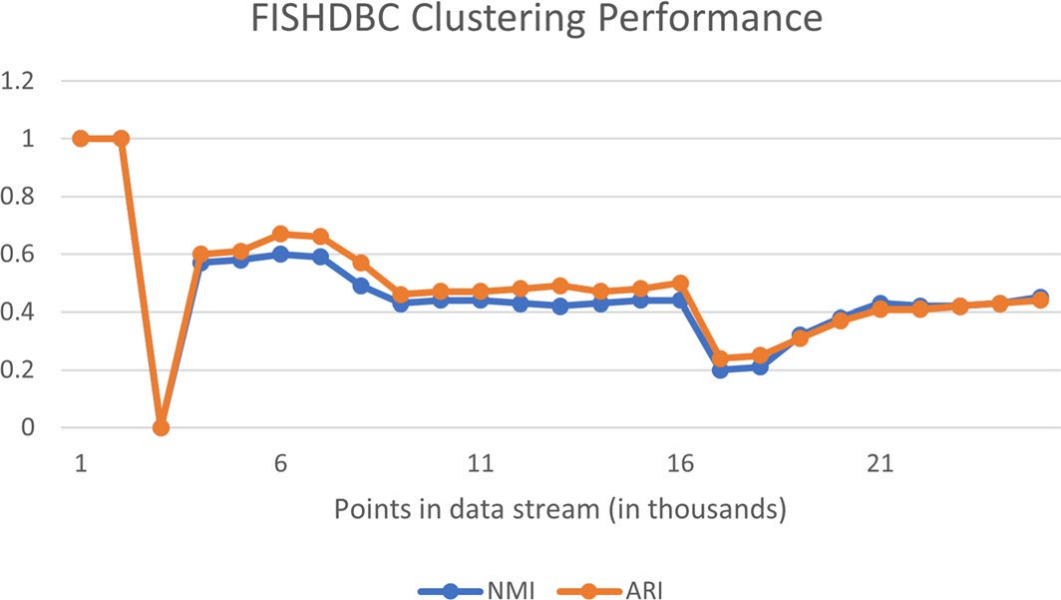
DEC loss during training

The performance of FISHDBC, WCDS and DEC are provided in this section. Figures 8 and 9 demonstrate the performance of the WCDS and FISHDBC algorithms respectively.

#### Evaluation and observations

The scores achieved by the DEC model are not surprising given the relative performance of the previous two models and fall within the overall range of the scores returned by FISHDBC and WCDS. Just looking at the NMI metric, we could make a case that DEC actually performs worse, since 0.498 is roughly a 10+% decrease from the 0.56 average of the other two. The ARI score fluctuates, so a clear baseline cannot be determined.

**Fig. 10:**
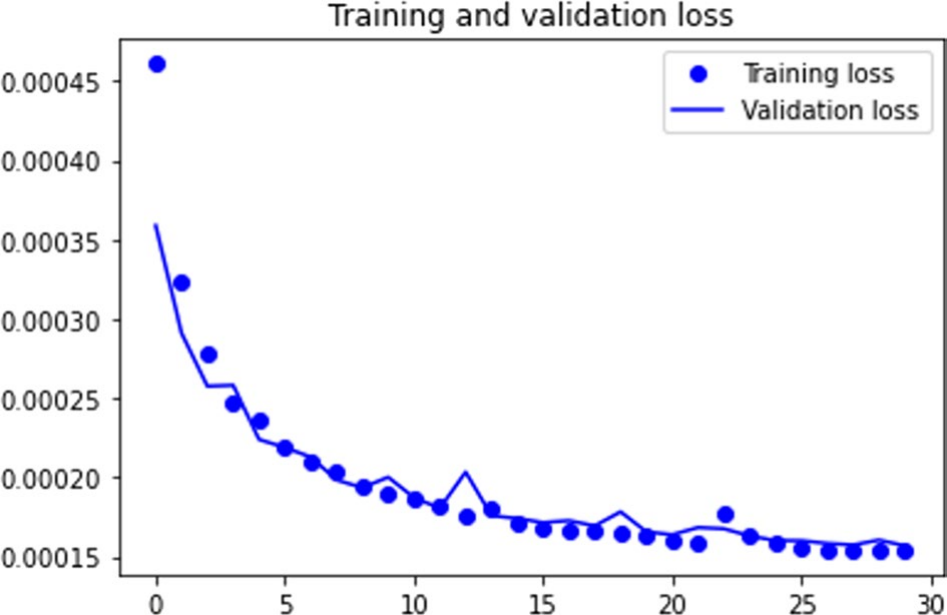
DEC loss during training

Despite being a powerful neural network, the DEC architecture has trouble processing the HAR data. We do know that the model is capable of reducing some knowledge based on the reported loss values (Fig. 10), but we assert that further feature reduction is necessary to obtain better clustering results. Several researchers achieved excellent performance on HAR data using just the accelerometer and gyroscope values [14, 32, 65], and since the MobiAct data also includes orientation data, the model should be capable of compressing the data in a way that excludes most of the orientation values. The quick drop in loss value and eventual stagnation imply that DEC easily reduces the feature set (perhaps by removing some orientation knowledge) in the first few epochs, but had difficulty in further feature compression. The model was able to quickly reduce the loss from 0.0359 to around 1.9e$-$04 and the clustering quality from the initial 0.357 NMI to a 0.493 NMI in just a few epochs.

**Table 2:** Summary of experimental results on MobiAct, UCI, and MNIST datasets

Algorithm	Dataset	Class. ACC	Clustering	
			ARI	NMI
*Stand-alone Clustering Algorithms*				
FISHDBC	MobiAct SLH	–	0.348	0.535
WCDS	MobiAct SLH	–	0.497	0.568
DEC	MobiAct SLH	–	0.406	0.498
*Autoencoders*				
DEC	MobiAct	29%	0.240	0.320
	UCI	49%	0.490	0.540
	MNIST	91%	0.910	0.910
2D CNN	MobiAct	93–99%	0.441	0.594
2D CNN + AE	MobiAct	–	0.340	0.555
LSTM AE	MobiAct	89%	0.344	0.598
	UCI	95%	0.461	0.613
*Hybrid Autoencoders*				
Hybrid AE	MobiAct (Fall)	94%	0.719 (K-Means)	0.856 (K-Means)
			0.678–0.832 (FISHDBC)	0.743–0.808 (FISHDBC)
	UCI	95%	0.990 (K-Means)	0.990 (K-Means)
			0.731–0.796 (FISHDBC)	0.870–0.912 (FISHDBC)
	12-Class MobiAct	95%	0.831 (K-Means)	0.897 (K-Means)
			0.860 (FISHDBC)	0.880 (FISHDBC)

We applied DEC to the MNIST dataset (see Table 2). To understand DEC’s performance on other non timeseries data, which showed gradual but continuous improvement in both loss and clustering results [48]. We can conclude two things: (1) the high performance of DEC on the MNIST(hand written digit) dataset compared to the relatively low performance on the MobiAct data shows the higher complexity inherent residing in HAR sequence data, and (2) the MobiAct data is capable of being compressed and reduced but requires additional methods of processing to further extract the hidden knowledge within the features.

On the HAR datasets, DEC returns final clustering scores of 0.54 NMI and 0.49 ARI for the UCI HAR dataset and 0.32 NMI and 0.24 ARI for the MobiAct data. Based on the literature [48] and our own experiments, DEC method is powerful, achieving 84% accuracy and 0.84 NMI on the non-timeseries MNIST dataset [48], and 91% accuracy and 0.91 NMI from our experimental results. Compared to the MNIST data, HAR classes (activities) naturally flow into each other, making it difficult for the algorithm to untangle the overlapping values into separate classes. Adding more data causes performance to drop even more. The results demonstrate that temporal feature extraction is required to effectively process time-series data.

### Performance of the enhanced AE feature extractors

We discuss the results of the different enhanced AE models below.

#### 2D-CNN with and without MLP AE

The 2D CNN model achieves 98–99% classification accuracy provided that prior to training, we upsample the lesser classes in the entire dataset using SMOTE (Synthetic Minority Over-sampling Technique) [63]. Without any sampling techniques, the classification accuracy drops to around 92–93%. Further experiments with upsampling just the training data and leaving the class distribution in the testing data untouched grants 94–95% classification accuracy. We believe that the third method is a more accurate representation of the real-life data. The upsampling increased the low number of observations for some activities, particularly for the brief transitional actions such as CSI (Car-Sit-In) and falling actions. Nevertheless, the sampled data does not accurately reflect the class distribution in real-life HAR data streams for where more standard actions, such as walking or standing outnumber the short transitional movements or any falling actions. Table 2 represents the 2D CNN classification results.

#### LSTM AE

Table 2 shows the resulting accuracy of the LSTM AE model, 72% after 150 epochs (out of 300). Additional epochs do not improve the accuracy score which is subpar compared to the previous methods. If classification accuracy is poor, then the chances that clustering performance on the same features being good are very low. Therefore, we have an incentive to maximize classification accuracy prior to clustering. The first change that is made to the architecture is replacing the LSTM layers with BiDirectional LSTM layers to enhance training. This alone improves accuracy by 8 percent. As a second improvement, we use LSTM classifier instead of a simple MLP.

The above changes improve the accuracy to 89% for the MobiAct dataset. Further experiments with model architecture do not improve the accuracy beyond this value. Clustering using these temporal features with K-Means grant us NMI and ARI scores of 0.489 and 0.310, respectively, and 0.598 NMI and 0.344 ARI with FISHDBC (Table 2).

#### Hybrid Conv AE + LSTM

With the Conv AE + LSTM hybrid architecture, we achieve a classification accuracy of 95%, and 0.831 ARI and 0.897 NMI for K-Means clustering. This is an improvement on the 0.523 ARI and 0.806 NMI when the full dataset has been used. The clustering results using FISHDBC are 0.86 ARI and 0.88 NMI, which show that the algorithm returns more consistent clusters compared to the previous scores of 0.73$-$0.79 ARI and 0.87$-$0.91 NMI. We observe that while classification accuracy does not improve, the clustering accuracy clearly does. This concludes that although the fall activities are minor in the grand scope of the dataset (3%), their impact on the interpretability of the cluster is quite substantial, showing that these minority actions do conflict with the other activities.

With the fall MobiAct dataset, our initial test using the hybrid model returns 93.5–94% classification accuracy. This number is improved to a definitive 95% when we introduce a 50% overlap in the data sequences. We, however, believe the underlying distribution between activities remains the same, and the increased performance is simply because the larger classes have become even larger.

The experiments using the UCI HAR dataset provides similar accuracy of around 94-95%, but this is achieved only after substituting BiDirectional LSTM layers for increased computational power. It is worth noting that we could not produce the reported performance(98% accuracy) of a similar model [34].

### Comparison of results for MobiAct vs UCI HAR

The features extracted from the UCI HAR dataset enable near-perfect clustering metrics (0.99 ARI and 0.99 NMI ) with K-Means, demonstrating the model’s ability to extract valid temporal features and form clear clusters. For the MobiAct dataset, K-Means achieves clustering scores of 0.719 ARI and 0.856 NMI, as shown in Table 2. The performance on UCI HAR is largely due to its simplicity, with only six standard activities such as walking, standing, and sitting, in contrast to MobiAct’s more complex 16 activities and 80,000 sequences.

The K-Means clustering performance using the 2D-CNN model, despite high classification accuracy, does not translate to superior clustering. Clustering directly on CNN activations results in 0.441 ARI and 0.594 NMI, and passing the activations through an AE slightly reduces performance to 0.340 ARI and 0.555 NMI. These results are similar to those obtained with statistical clustering methods, as shown in Table 2.

Further improvements in model architecture do not significantly enhance clustering performance. Notably, applying this model to segmented MobiAct data causes memory issues, leading to process crashes without generating results.

### Results with temporal extraction

Two additional temporal feature processing methods were tested to further improve the clustering results as discussed below.

**Table 3:** Time taken to process a batch of 128 sequences

Architecture	Time
Time-distributed	21 s
Tri-Head Model	48 s

We see that the accuracy performance using the Tri-Head TE model only gains a slight improvement over the regular Time-distributed TE model, going from 93.3 to 93.8% on the raw MobiAct dataset. Accordingly, the engineered data provides similar scores of 93.6% for the Time-distributed model against the 93.8% of the Tri-Head architecture. For both the Tri-Head and Time-distributed designs, the full sequence MobiAct returns high classification accuracy. Seemingly, both models are practically identical in terms of classification, which raises the question whether the more powerful and subsequently resource hungry Tri-Head implementation is necessary for HAR feature extraction when the simpler model performs just as well and with more efficiency. As shown in Table 3, the time taken to process a sequence using the Tri-Head model is more than twice the amount taken by the previous iteration at around 48 s.

However, the clustering performance between the two designs shows a different story, as Table 4 displays the clear increase in metric scores for the Tri-Head model across all the datasets. From this, we can conclude that the newest iteration of TE is capable of extracting more applicable knowledge for clustering algorithms at the cost of greater resource draw. Presumably, the use of three levels of analysis on each sequence yields features that are more easily distinguishable by the simple distance metrics that clustering algorithms use. We can notice this fact reflected in the slight performance increases in the Tri-Head classification accuracy scores—in the overall picture, the additional knowledge might not affect weight changes in the TE model greatly, but if taken into the context of distance metrics, the change is quite significant.

**Table 4:** Performance comparison of temporal feature extractors using K-Means and FISHDBC clustering

Feature extractor	Dataset	Classification ACC	ARI	NMI
*Base and improved temporal extraction*				
Base TE	Many-to-One MobiAct	92.6%	–	–
Base TE	Seq2Seq MobiAct	92.7%	–	–
Base TE	UCI	92.4%	–	–
Improved TE	Full MobiAct	95.0%	–	–
*Time-distributed temporal extraction*				
TimeDist. TE	Raw Reduced MobiAct	93.1%	0.628 (K-Means)	0.664
			0.372 (FISHDBC)	0.564
TimeDist. TE	Eng. Reduced MobiAct	93.6%	0.431 (K-Means)	0.649
			0.348 (FISHDBC)	0.543
TimeDist. TE	Full MobiAct	98.0%	0.990 (K-Means)	0.990
			0.980 (FISHDBC)	0.980
TimeDist. TE	UCI	92.2%	0.927 (K-Means)	0.929
			0.875 (FISHDBC)	0.860
Tri-head temporal extraction				
Tri-Head TE	Raw Reduced MobiAct	93.8%	0.522 (K-Means)	0.749
			0.648 (FISHDBC)	0.634
Tri-Head TE	Eng. Reduced MobiAct	93.8%	0.548 (K-Means)	0.784
			0.673 (FISHDBC)	0.684
Tri-Head TE	Full MobiAct	98.0%	0.990 (K-Means)	0.990
			0.980 (FISHDBC)	0.980
Tri-Head TE	UCI	93.5%	0.990 (K-Means)	0.990
			0.930 (FISHDBC)	0.940
Tri-Head TE	512-win MobiAct	93.8%	0.579 (K-Means)	0.821
			0.540 (FISHDBC)	0.681

### Effect of using full vs segmented data

When deploying our pipeline for real-time clustering, balancing model performance with computational cost is crucial. Our experiments show that using the Full Sequence MobiAct data yields near-perfect scores, particularly when using Time-distributed models. This high performance is likely due to giving LSTM models access to more time steps, allowing them to learn detailed temporal dependencies for each activity. Additionally, replacing LSTM layers with BiLSTM layers, which capture both past and future time steps, further enhances accuracy.

However, using Full Sequence data is not realistic for real-time applications, where the system needs to make predictions quickly, often every second. Models trained on long sequences (e.g., sequences up to 5 min) may not perform well on shorter, real-time sequences. Ideally, we want to replicate the performance seen with Full Sequence data but using shorter, more practical sequence lengths.

One approach to this is increasing the window size. The Full Sequence data can be thought of as having a very large window size, where all activity data for a long period is treated as a single sequence. Our experiments tested different window sizes to balance performance and practicality. For example, increasing the window size from the default 200 time steps (1 s) to 512 time steps (2.56 s) resulted in similar classification accuracy (93.8%) with the Time-distributed architecture. However, clustering performance improved significantly, with K-means achieving ARI and NMI scores of 0.579 and 0.82, and FISHDBC reaching 0.540 ARI and 0.681 NMI.

Interestingly, increasing the window size to 1024 time steps did not yield further improvements and even led to a decrease in performance. One reason for this might be that larger windows can cut across activities in a way that creates mixed sequences. For instance, if a sequence contains 800 time steps of walking, 300 of falling, and 400 of lying down, a 1024-window might split the falling activity in half, causing the model to incorrectly classify the mixture based on the dominant activity (e.g., walking).

Another observation from our experiments is that processing time per batch decreases as the window size increases. This could be because larger windows allow the model to process fewer, but longer, sequences in parallel, reducing the overhead of frequent sequence transitions.

### Ablation study

We conducted an ablation study to assess the contribution of each architectural component across datasets. Standalone clustering algorithms (FISHDBC, WCDS, DEC) yielded limited performance on MobiAct (e.g., ARI 0.348–0.497), indicating challenges in unsupervised modeling of raw sensor data. Autoencoder-based models, including LSTM-AE and CNN+AE, provided moderate gains, but still underperformed (ARI $\le$ 0.461) due to limited temporal or spatial context modeling. Our proposed hybrid architecture, which integrates CNNs, LSTMs, and embedding projection, significantly improved clustering outcomes, achieving ARI/NMI up to 0.831/0.897 on MobiAct and 0.99/0.99 on UCI. The Tri-Head Temporal Encoder further enhanced robustness across reduced feature sets. These results confirm that combining spatial and temporal encoding is critical for learning cluster-friendly embeddings in high-dimensional HAR data and demonstrate the superiority of our architecture over isolated components.

## Visualization

TMAP (Tree MAP) [61] is a modern contribution in the high-dimensional reduction and data visualization domain. The authors proposed the algorithm in 2020 for the purpose of displaying extremely large dimensional data commonly found in molecular chemistry studies. They were motivated due to the need to effectively and clearly display the relationships between complex features in a method that is still interpretable. TMAP allows visualizations in the form of an aesthetically pleasing connected tree.

It first approximates a k-nearest neighbour graph, then the algorithm evolves the graph into a minimum spanning tree (MST). Any cycles in the graph are pruned, effectively lowering computational complexity for low-dimensional embeddings. To reduce effort required to graph potentially millions of vertices in the tree, another approximation-based method is used to draw the MST. The result is a visually appealing graph displaying the dataset’s low-dimensional representation.

**Fig. 11:**
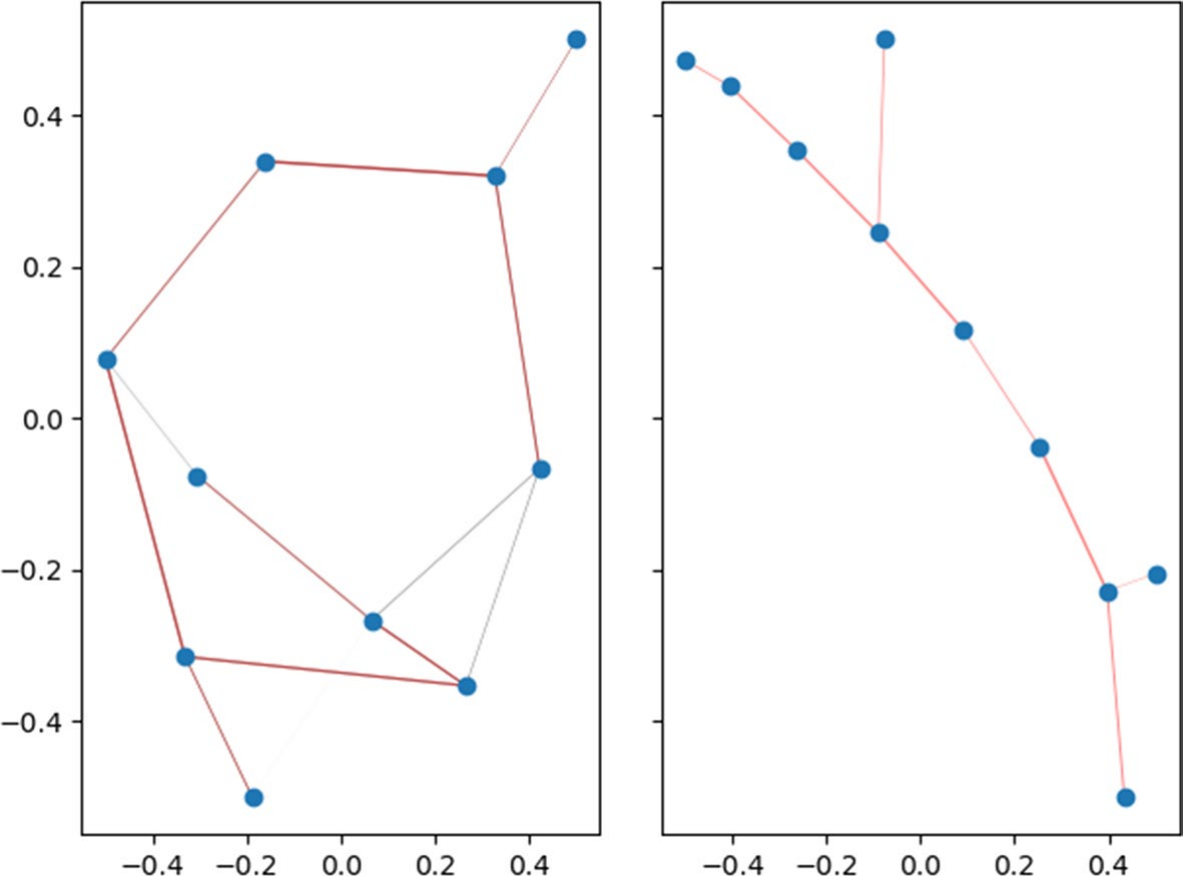
The tree structure created and idealized by TMAP [61]

Figure 11 shows an example of the MST created by TMAP and how the algorithm re-configures the information for display. An interactive webpage is returned, which allows for exploration of the graph.

**Fig. 12:**
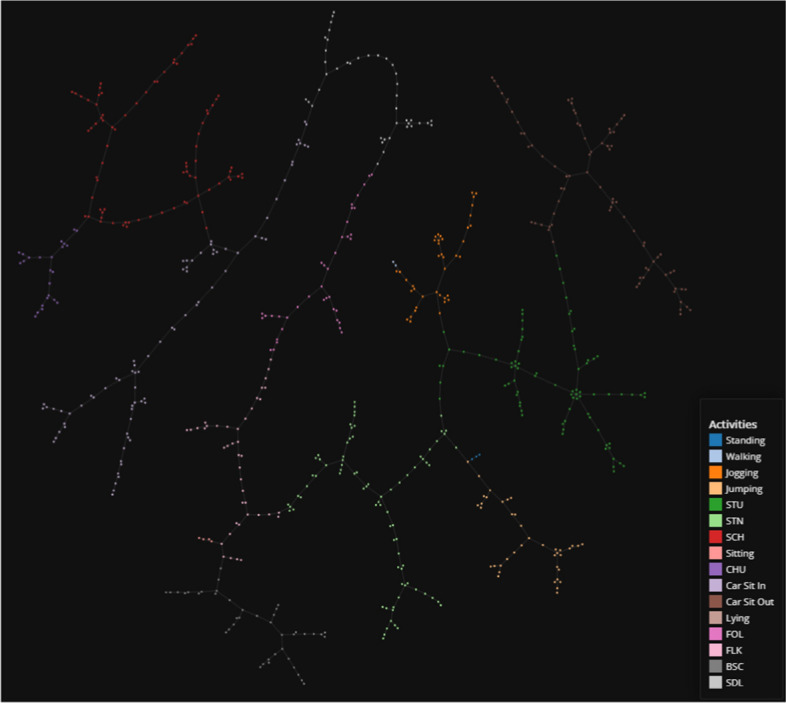
TMAP plot of the full sequence MobiAct dataset used in the LSTM + AE

**Fig. 13:**
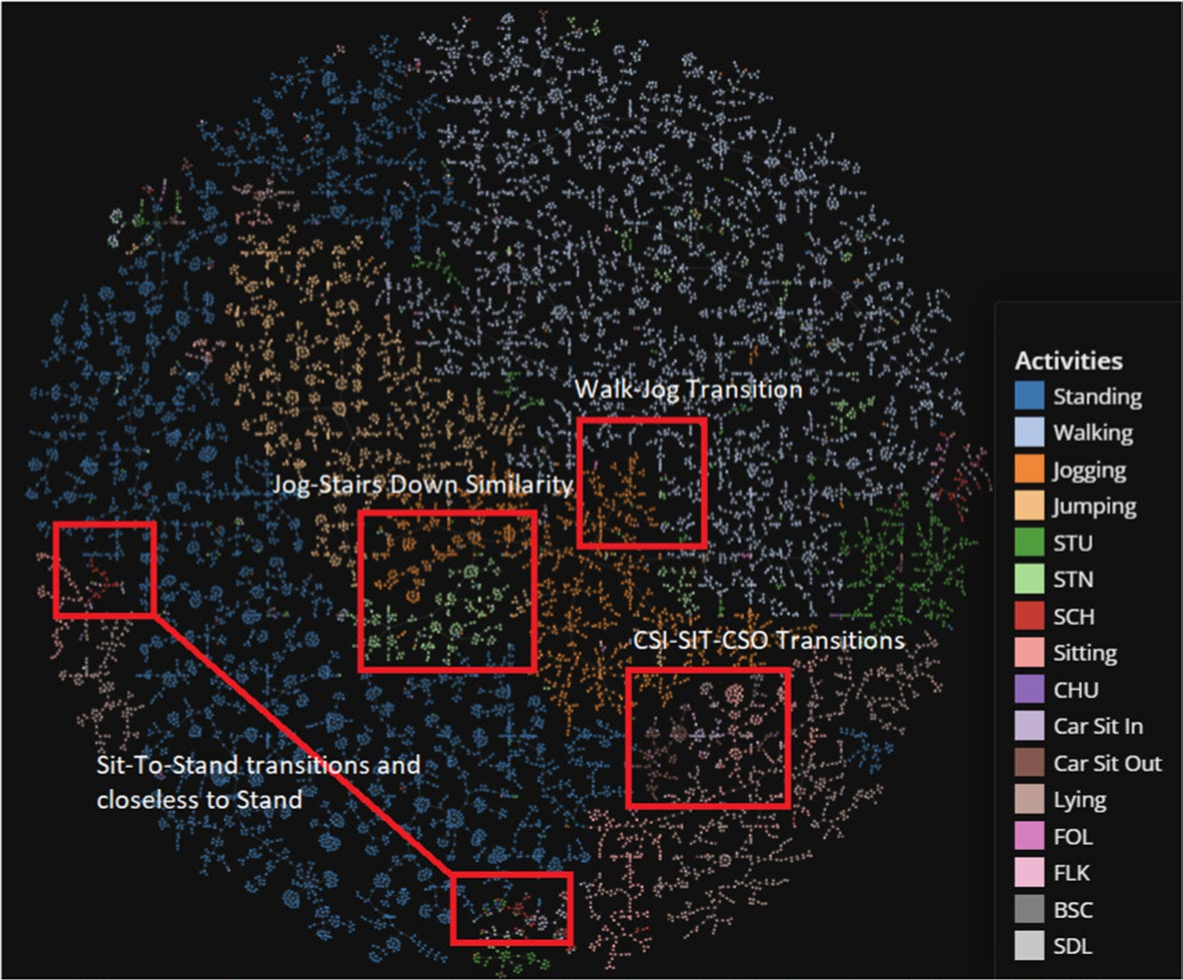
TMAP plot of the segmented MobiAct dataset used in the LSTM + AE. Annotations displaying patterns are added to the plot

We apply TMAP on the two HAR datasets to determine if any intuitive knowledge can be inferred from the cluster results. Figure 12 shows the full sequence MobiAct plotted using TMAP. Given the high performances returned by the LSTM + AE model for both classification and clustering, it is not surprising to see the visual clusters are clear and distinct, supporting the fact that supervised learning in combination with long temporal sequencing is capable of extracting excellent features. We also include the TMAP graph for the segmented MobiAct dataset as a comparison (Fig. 13). The annotations added to Fig. 13 highlight some of the intuition we can extract from visualizing the dataset. For example, we see understandable connections between activities such as WAL and JOG, and the natural transition triplets of CSI, SIT, and CSO.

**Fig. 14:**
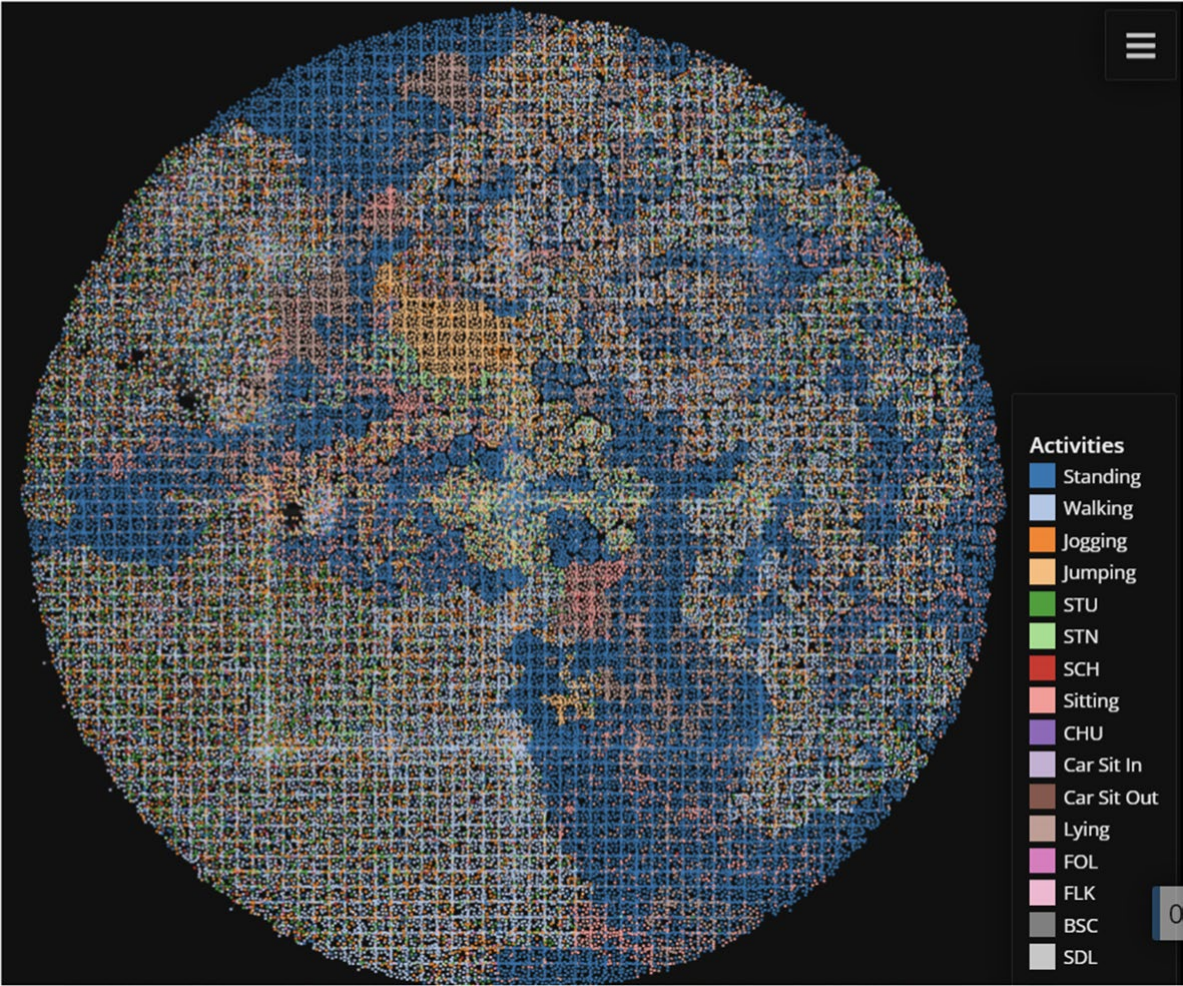
TMAP plot of the entire raw MobiAct dataset

Immediately, we can understand how effective temporal feature extraction is when we compare the TMAP plots of the original MobiAct (Fig. 14) and the temporal-reduced MobiAct. Where the original MobiAct graph has the prominent actions (WAL and STD, etc.) dominate most of the space with the less frequent activities weaved in-between, the temporally-extracted plots show distinct clusters between most of the activities. In the full sequence data, the classes are more distinct since the clustering performance is better overall while the raw data plot still provides clear relationships between the activities despite slightly-poorer cluster scores.

**Fig. 15:**
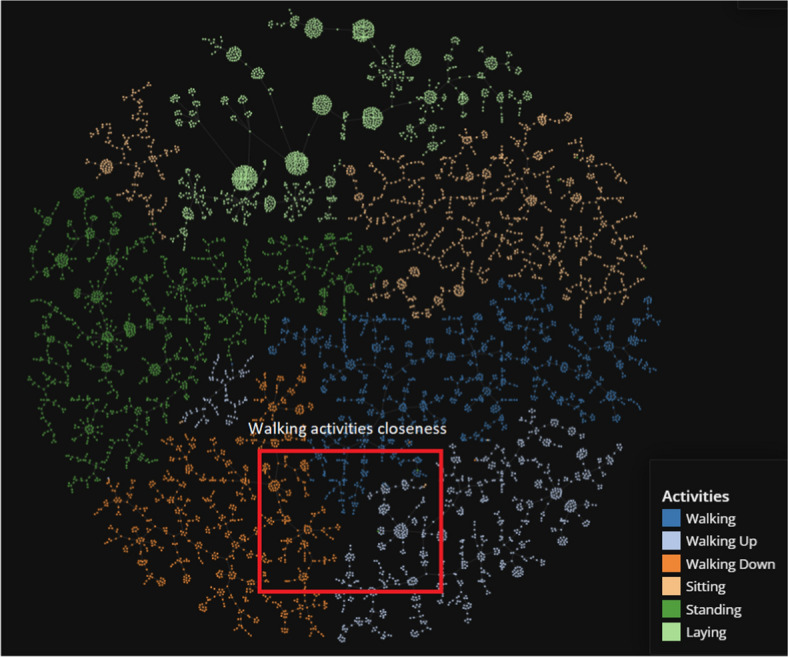
TMAP plot of the UCI HAR dataset with annotations

Figure 15 displays the result of applying the TMAP algorithm to the UCI HAR clusters returned by the LSTM AE model. The clustering and classification result were also high, so the relatively clear segmentation of the clusters help explain the model’s performance. The slight mixing of different coloured points correspond to a drop in clustering scores. Similar to the MobiAct visualization, we can extract intuition regarding the dataset from examining the graph. The dataset is significantly ’easier’ compared to the MobiAct dataset, hence the clean separations and clear relationships between actions. We can clearly observe the similarities and intuitive features shared among the walking actions. This pattern between walking activities was previously noticed in the MobiAct dataset as well.

## Conclusion

Clustering highly complex streaming HAR data is limited by the decreased computational capabilities in favour of increased computational speeds in simple clustering algorithms. Autoencoder models have powerful architectures that are capable of learning and extracting the deep feature representations of datasets. These reduced representations can be used to return meaningful clusters for further analysis. We chose AE after conducting a comparative study of various existing models. We implement multiple AE architectures, use classification with supervised learning to train different feature extractor models, and then use the trained model with unsupervised clustering. Experiments were conducted with multiple data sets having different numbers of activities. We conclude that effective processing of streaming complex HAR data must utilize temporal feature processing in combination with deep supervised learning. Cluster visualization via the TMAP algorithm provides further insights into the strengths of temporal-based feature extraction and allows for intuitive exploration of the returned clusters.

*Limitations*: Despite the promising results and the comprehensive methodology proposed in this study, several limitations should be acknowledged to contextualize the findings and guide future research. Our experiments were primarily conducted on benchmark datasets such as MobiAct and UCI-HAR, including an engineered subset (MobiAct SLH). However, more complex and unstructured datasets were not included due to our focus on transitional activities and multiphase model development with explainability. Additionally, although the final clustering stage is unsupervised, the feature extraction models rely on supervised training, which introduces a degree of label dependency. This highlights the need to explore self-supervised or contrastive learning methods for building a fully unsupervised clustering framework. The use of fixed-size sliding windows without overlap, while effective for segmenting sensor streams, may not fully capture transient or overlapping activities. Furthermore, while our proposed methods demonstrate robustness on existing datasets, scalability to more complex sensor environments-such as multimodal sensor arrays or longer time horizons-has not been empirically tested. Another limitation lies in the lack of a comprehensive evaluation of computational costs across embedded devices, which would provide valuable insight into the feasibility of real-time deployment in resource-constrained environments.

*Future work*: We plan to expand dataset diversity by incorporating more complex and real-world HAR datasets such as Opportunity [66] and Hang-Time HAR [60] to improve generalizability. We will also explore replacing supervised feature extraction with self-supervised or contrastive learning methods to better align with the unsupervised nature of clustering. In addition, we aim to capture short-duration and transitional activities more effectively through adaptive temporal modeling techniques. To improve scalability for high-dimensional sensor data, we will investigate attention mechanisms and graph-based learning for efficient modeling of spatial dependencies. Finally, we will conduct detailed runtime and resource profiling across hardware platforms to assess the feasibility of real-time deployment. These directions aim to further enhance the robustness, scalability, and real-world applicability of HAR clustering frameworks.

## Data Availability

The UCI HAR dataset is available at (https://archive.ics.uci.edu/dataset/240/human+activity+recognition+using+smartphones). The MobiAct V2.0 dataset is available upon request from the BioMedical Informatics & eHealth Laboratory (https://bmi.hmu.gr/the-mobifall-and-mobiact-datasets-2/). The dataset was provided to us after signage of a database usage agreement which establish the terms and conditions of data usage.
